# Is Partial Nephrectomy A Primary Option for Patients with T1b Renal Cell Carcinoma—A National Population-Based Study

**DOI:** 10.5152/tud.2025.24081

**Published:** 2025-04-04

**Authors:** Sven Lundstam, Tarik Almdalal, Andreas Karlsson Rosenblad, Börje Ljungberg, Committee National Swedish Kidney Cancer Register Steering

**Affiliations:** 1 Departments of Urology and Oncology, University of Gothenburg Sahkgrenska Academy, Gothenburg, Sweden; 2 Department of Surgery and Urology, Eskilstuna County Hospital, Eskilstuna, Sweden; 3 Regional Cancer Centre Stockholm-Gotland, Stockholm, Sweden; 4 Department of Statistics, Uppsala University, Uppsala, Sweden; 5 Division of Clinical Diabetology and Metabolism, Department of Medical Sciences, Uppsala University, Uppsala, Sweden; 6 Division of Family Medicine and Primary Care, Department of Neurobiology, Care Sciences and Society, Karolinska Institutet, Stockholm, Sweden; 7 Department of Surgical and Perioperative Sciences, Urology and Andrology, Umeå University, Umeå, Sweden

**Keywords:** cT1b, partial nephrectomy, pT1b, radical nephrectomy, RCC type, renal cell carcinoma, survival, tumor size, tumor stage

## Abstract

**Objective:**

Renal cell carcinoma (RCC) patients in clinical T1 RCC generally exhibit a favorable prognosis. Guidelines recommend partial nephrectomy (PN), also for cT1b RCCs. Despite a favorable prognosis, there remains risks for upstaging and recurrence for cT1b RCC patients, and the preference for PN has been questionable. Clinical and morphological variables and overall survival (OS) were characterized in a national real-world population.

**Methods:**

Data from the the National Swedish Kidney Cancer Register 2005-2014, with non-metastatic cT1bRCC patients treated surgically and having ≥5 years potential follow-up were included (n = 2006). Patients gender, age, stage, tumor size, RCC type, local and distant tumor recurrence were evaluated.

**Results:**

Among 2006 patients (1219 males, 787 females; mean age 66 years), 1705 underwent radical nephrectomy (RN), and 301 PN. Upstage from cT1b to pathological T3a occurred in 304 (15%) patients. Recurrent disease was diagnosed in 318 (16%) patients, with higher rates in pT3a (25%) compared to pT1b (14%). There was no significant difference in disease recurrences observed between the surgical techniques. Factors associated with increased recurrence risk included age, T-stage, N-stage, and tumor size, while papillary and chromophobe RCCs were associated with decreased risk. Patients with pT3a RCC had a worse 5-year OS rate (67%) compared with pT1b (83%; *P *< .001, log-rank test). In adjusted analyses, age, tumor size, pT-stage, and pN-stage were associated with OS, while treatment with PN was non-inferior compared with RN (hazard ratio 0.91, 95% CI: 0.71-1.45, *P* = .431).

**Conclusion:**

Patients with clinical T1b RCCs face a non-negligible risk for tumor upstaging, disease recurrence, and decreased OS. The adjusted analyses showed that PN was non-inferior to RN, supporting the recommendation to offer PN.

**Main Points**

- Patients with clinical T1b RCCs have a non-negligible risk for tumor upstaging, disease recurrence, and decreased OS.
- Tumor size increase and upstaging from clinical T1b to pathological T3a were factors afflicted with worse prognosis.
- The adjusted analyses showed that PN was non-inferior to RN supporting the recommendation to offer PN.

## Introduction

Renal cell carcinoma (RCC) incidence has slowly been rising over the last decades, mostly attributable to an increase in abdominal imaging and increased detection of asymptomatic renal masses.^1^ Presently, more than 60% of the RCCs are diagnosed incidentally, which has brought on generally lower tumor stages.^2^ By this shift to lower stages, currently more than 60% are T1 RCC, being in the stage with the lowest tumor progression. The treatment of patients with T1 RCCs therefore has changed from radical nephrectomy (RN) to nephron-sparing treatments. Currently, guidelines recommend nephron-sparing treatment to preserve maximum kidney function when feasible.^3^,^4^ Patients with clinical T1b RCC, however, exhibit a higher incidence of pathological upstaging than pT1a and have a higher risk for metastases.^5^ Since recommendations for ablative treatments are limited to cT1a, surgery is the treatment option for most patients with cT1b^3^ Partial nephrectomy (PN) is more complex and challenging and is afflicted with higher risks of leaving tumor cells behind compared with RN, which includes removal of the entire kidney within the Gerota’s fascia. This risk is especially high in patients with local tissue and vein invasion.^3^,^4^,^6^,^7^ Positive surgical margins are discovered in 7%-10% after PN, which is associated with recurrent disease that may impair survival.^8^^-^^10^ However, oncological outcomes were shown to be similar between PN and RN for T1b RCC patients.^3^,^11^ Thus, the optimal treatment for patients with cT1b RCC, considering oncological outcomes and renal function, remains unclear.

## Aim

The aim of this study was to assimilate the outcomes of patients with cT1b RCC in terms of recurrence and overall survival (OS) treated with RN or PN in a real-world setting.

## Material and Methods

### Participants and Eligibility Criteria

The present study included all surgicallytreated patients with primary non-metastatic cT1b RCC having a potential follow-up *time* of more than 5 years, registered prospectively in the the National Swedish Kidney Cancer Register (NSKCR) between January 2005 and January 2014. The 2006 patients had a mean age of 66 years at, 1219 (61%) males and 787 (39%) females (Table 1).

### Study Design and Outcome Measures

After 5 years follow-up, the patients were retrospectively evaluated by their urological department to determine whether any recurrence had occurred during the follow-up time. Recurrence-free survival timewas measured from thedate of treatment to the dateof recurrence. The ´Swedish National Population Register was used for information on OS. Survival time was calculated from the date of treatment to the date of any cause of death or censored if alive at the end of December 2020. Information on clinical data and primary surgery was extracted from NSKCR. The seventh TNM stage and the 2016 WHO classification were used for TNM stage and RCC type classification, respectively.^12^,^13^ The largest diameter of the tumors on the preoperative computerized tomography defined the tumor size.Part of this patient cohort was included in a previous study.^5^

### Ethical Approval

The study was approved by the regional Ethical Review Board of Northern Sweden (Dnr 2012-418-31M) and the Swedish Ethical Review Agency (Dnr 2019-02579).

### Statistical Analyses

Categorical data are shown with numbers and percentages. Ordinal and continuous data are shown as means ± SDs. Pearson’s *χ*^2^-test was used between groups and the Mann–Whitney *U* test for continuous variables. The Kaplan–Meier method was used to construct survival curves and tested with the log-rank test. Cox proportional hazards regression tests were done for unadjusted and adjusted analyses with time-to-death as the outcome. Results are reported as hazard ratios (HRs) and accompanying 95% CI. For statistical analyses, IBM SPSS Statistics 26.0 (IBM SPSS Corp.; Armonk, NY, USA) was used with 2-sided *P*-values <.05 regarded as significant.

## Results

Of the 2006 included patients, RN was performed in 1705 (85%) and PN in 301 (15%) of the patients (Table 1). Most patients (n = 1625, 81%) had clear cell RCC (ccRCC), while 232 (12%) patients had papillary RCC-type (pRCC), 115 (6%) chromophobe RCC-type (chRCC), and the remaining 34 (2%) had other RCC types.

Among the 2006 patients with cT1b, 303 (15.1%) were upstaged to pT3a, including 250 (15.4%) ccRCCs, 27 (11.6%) pRCCs, 20 (17.4%) chRCCs, and 6 (17.6%) patients with other RCC types. The occurrence of recurrences was observed among 281 (16.5%) of the RN-treated patients and 37 (12.3%) of the PN-treated patients. Notably, 279 (99.3%) of the RN-treated patients with tumor recurrence experienced metastases, compared with 22 (59.5%) of the PN-treated patients (Table 1).

In total, recurrent disease during ≥5 years follow-up, including local recurrences and distant metastases, was diagnosed in 318 patients (15.9%), including 281 (17.3%) ccRCCs, 25 (10.8%) pRCCs, 5 (4.3%) chRCCs, and 7 (20.6%) patients with other RCC types (not in table). Among patients with recurrent disease, 238 (14%) had pT1b and 80 (25%) patients were upstaged to pT3a (not in table). The lung was the most common recurrence site (50.6%), as shown in Table 2. Among the 318 patients with recurrent disease, 95 (30.0%) had 2 or more metastatic sites (Table 2). Most patients (15 of 17) with local recurrence were primarily treated with PN while 2 RN patients had local kidney groin recurrence. Patients’ age at diagnosis, stage upgrade to pT3a, N-stage, and tumor size all had a significant independent association with increased risks of recurrent disease, while patients having pRCCs or chRCC had decreased risks (Table 3).

**Figure 1.**
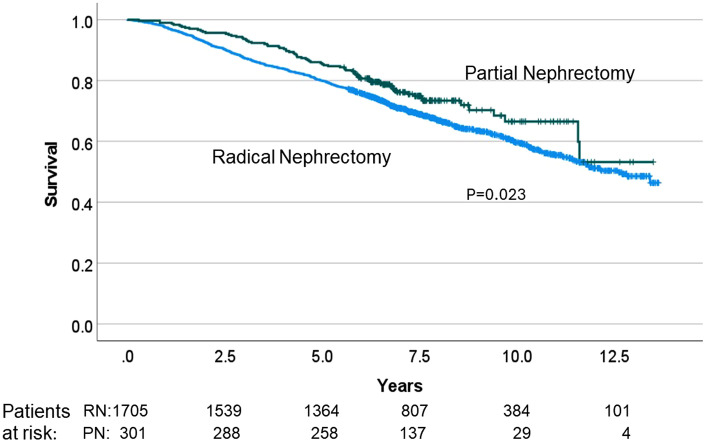
Kaplan–Meier curves of univariate overall survival probability in relation to PN vs. RN in 2006 patients with clinical T1b renal cell carcinoma. Number of patients at risk are shown below the corresponding time points.

Patients with pT3a RCC had significantly lower (67%, *P* < 0.001) 5-year OS rate compared to those with pT1b (83%, data not shown). Partial nephrectomy patients had a significantly better OS (*P* = .023) than RN-treated patients (Figure 1). In adjusted analyses, age, pT-stage, cN-stage, and tumor size remained significantly associated with OS, while PN compared with RN had a non-different association (HR 0.91; 95% CI: 0.71-1.16, *P* = .431) (Table 4).

## Discussion

It was shown, after adjusted analyses, that surgical treatment with PN entailed a non-inferior OS compared with RN, in a nationwide cohort of real-world patients with clinical T1b RCC. This non-differential survival was obtained despite the significantly higher risk of positive surgical margins when performing PN due to leaving unrecognized local tumor tissue and vein invasion.

Generally, RCC patients without metastases in clinical stage T1 at diagnosis have the best prognosis among the RCC stages.^3^ However, despite this advantageous prognosis, there remains a risk for progressive disease.^5^ This is partly due to the risk of pathological tumor upstage to pT3a in patients with cT1b RCC, who are at a higher risk for local and metastatic recurrent disease.^5^,^14^^-^^16^ Radical nephrectomy has an advantage for these patients with pathological upstage, lowering the risk of leaving tumor tissue behind compared with PN. Improved preoperative imaging is desirable to enhance the detection of upstaging and to guide surgery planning.^17^,^18^ It is well-known that local recurrence implies reduced survival, while nephron-sparing aligns with fewer cardiovascular events.^3^,^19^ Partial nephrectomy with more remaining nephrons is advocated in patients with already reduced kidney function, especially to prevent any need for hemodialysis. Also, in newly diagnosed RCC patients with normal baseline serum creatinine levels, 26% had a glomerular filtration rate ≤60 mL/min.^20^ Therefore, PN is an advised option for oncological outcomes and preserving kidney function in patients with cT1 RCC.^3^,^21^

Performing PN is more intricate than RN and has a higher incidence of complications.^3^,^22^ It has been debated whether PN enhances OS compared with RN due to more preserved renal function. Several retrospective analyses have suggested increased cardiovascular mortality and decreased OS for RN-treated patients compared to those treated with PN. In some series, this survival difference was found only for patients with comorbidity.^23^ In a systematic review by Chung et al,^24^ the authors reported significantly improved OS in PN-treated compared to RN-treated patients (HR 0.74; 95% CI 0.75-0.95). In contrast, the significant advantage in OS for PN found in univariate analysis in our study disappeared in the adjusted analysis, showing a similar risk for reduced OS in PN-treated patients compared with RN-treated. Our data confirm results from 2 studies using meta-analyses showing similar RFS and OS rates.^21^,^25^ Furthermore, we found that patients treated with PN had similar risks for disease recurrence as patients treated with RN. This unexpected equality might be due to the tumor biology irrespective of type of performed surgery but also due to the patient’s treatment being based on several considerations judged by individual surgeons such as tumor location, complexity, and tumor size, as well as the patient’s preferences.^3^,^4^ Moreover, morphologic RCC type remained, after adjusted analyses, a significant factor for both disease recurrence and OS. Patients with chRCC had significantly fewer occurrences of recurrences and showed a 58% lower risk for reduced OS than ccRCC, while patients with pRCC had a 46% lower risk for disease recurrence. These results are in line with those found in earlier studies.^5^,^26^

Furthermore, we showed that increased tumor size raised the risk for reduced OS by 1.8% per mm increase in tumor size. Thus, we show that tumor size is important for survival even in T1b RCCs, corroborating the outcome of prior research.^5^,^27^ Using the SEER cancer database, Tang et al also showed importance of tumor size for both overall mortality and cancer-specific mortality.^28^ The independent risks for tumor recurrence and lower OS by increasing tumor size clearly indicate that early tumor treatment is desirable even in patients with cT1b RCC.

### Strengths and Limitations

Limitations of the present study were that data on comorbidities, performance status, and cancer specificwere unavailable. Another limitation was that the NSKCR data was registered from several hospitals, which is why uniformity in data collection might have been unattainable and may have resulted in register discrepancies. However, when the register was validated, it had high equivalence and quality.^29^ The treatments of the patients were ultimately based on surgeons' and patients’ preferences. An important strength is that the patients represented a nationwide cohort, including practically all RCC patients nationwide.

It was shown that there was no statistically significant difference in OS between patients with cT1b-RCC treated with PN or with RN. Age at diagnosis, upstaging from cT1b to pT3a RCC, RCC type, and tumor size all were associated with OS. Furthermore, age, T-stage, RCC-type, and tumor size were associated with the occurrence of recurrent disease. The recommendation to offer PN to patients with cT1b RCC is supported by the results.

## Members of the Study Group:

The members of the National Swedish Kidney Cancer Register Steering Committee are given in the Appendix.

## Figures and Tables

**Table 1.** Characteristics of the 2006 Patients with Clinical T1b Renal Cell Carcinoma Included in the Study, in Relation to Performed Surgical Treatment

Variable	All Patients	Radical Nephrectomy	Partial Nephrectomy	*P*
	n = 2006	n = 1705	n = 301	
	100%	85.0%	15.0%	
Male, n (%)	1219 (60.8)	1002 (58.8)	217 (72.1)	**<.001**
Female, n (%)	787 (39.2)	703 (41.2)	84 (27.9)	
Age at surgery (years), mean (SD)	66.1 (11.7)	66.4 (11.6)	64.3 (11.8)	**.010**
pT-stage, n (%)				**<.001**
pT1b	1702 (84.8)	1426 (83.2)	276 (91.7)	
Upstaged p3a	304 (15.2)	279 (16.4)	25 (8.3)	
N-stage, n (%)				N/A
N0	1972 (98.3)	1672 (98.1)	300 (9.7)	
N1	34 (1.7)	33 (1.9)	1 (0.3)	
Tumor size (mm), mean (SD)	55.8 (8.6)	52.5 (7.5)	56.3 (8.7)	**<.001**
RCC type, n (%)				**<.001**
Clear cell RCC	1625 (81.0)	1419 (71.8)	206 (68.4)	
Papillary RCC	232 (11.6)	164 (9.6)	68 (22.6)	
Chromophobe RCC	115 (5.7)	93 (5.5)	22 (7.3)	
Other RCC types	34 (1.7)	29 (1.7)	5 (1.7)	
Tumor recurrence^a^, n (%)	318 (15.8)	281 (16.5)	37 (12.3)	N/A
Local recurrence	17 (5.3)	2 (0.7)	15 (40.5)	
Metastases	301 (94.7)	279 (99.3)	22 (59.5)	

N/A, not applicable; RCC, renal cell carcinoma; SD, standard deviation.

^ a^Tumor recurrence refers to observed recurrence during follow-up.

**Table 2.** Distribution of Sites of Recurrent Disease in Relation to Surgical Treatment in 2006 Patients with cT1bM0 Renal Cell Carcinoma at Primary Diagnosis

Site of Recurrence	Total	RN	PN
	n = 318	n = 281	n = 37
All sites	440	386	54
Lung	161	143	18
Skeletal	73	68	5
Lymph nodes	48	46	2
Treated kidney	20	0	20
Local groin	28	28	0
Adrenal	24	22	2
Contralateral kidney	9	9	0
Liver	36	32	4
Brain	10	10	0
Other	28	25	3

Among the 318 patients with recurrent disease, 95 (29.9%) had more than 1 site registered.

**Table 3.** Results for Cox Regression Analysis of Factors Important for Time to Diagnosis of Recurrence in 2006 Patients with Clinical T1b Renal Cell Carcinoma Adjusted for Age, Gender, Upgraded pT3a, N-Stage, RCC Type, Tumor Size, and Performed Surgery (PN vs. RN)

Predictor	Unadjusted			Adjusted^a^		
	HR	95% CI	*P*	HR	95% CI	*P*
Age (year)	1.024	1.014-1.035	**<.001**	1.023	1.012-1.034	**<.001**
Female vs. male	0.839	0.667-1.056	.135	0.793	0.628-1.002	.052
pT3a vs. pT1b	2.239	1.737-2.884	**<.001**	1.967	1.520-2.546	**<.001**
N1 vs. N0	6.971	4.314-10.943	**<.001**	1.343	1.120-1.611	**.001**
RCC type						
ccRCC	Ref.			Ref.		
pRCC	0.608	0.404-0.915	**.017**	0.551	0.359-0.846	**.006**
chRCC	0.234	0.097-0.566	**.001**	0.250	0.103-0.607	**.002**
Other	1.363	0.644-2.885	.419	1.500	0.708-3.180	.290
Tumor size (mm)	1.029	1.016-1.042	**<.001**	1.024	1.011-1.037	**<.001**
PN vs. RN	0.716	0.508-1.009	.056	0.896	0.626-1.284	0.551

Significant *P*-values are given in bold.

HR, hazard ratio; PN, partial nephrectomy; Ref., reference category; RN, radical nephrectomy. ^a^Adjusted for: age at surgery (years), female vs. male sex, pT1b vs. upgraded pT3a, RCC type, tumor size (mm), and surgery PN vs. RN.

**Table 4.** Results for Cox Regression Analysis on Overall Survival in 2006 Patients with Clinical T1b Renal Cell Carcinoma Adjusted for Age, Gender, Upgraded cT1b to pT3a, RCC Type, Tumor Size, and Performed Surgery (PN vs. RN)

Predictor	Unadjusted			Adjusted^a^		
	HR	95% CI	*P*	HR	95% CI	*P*
Age (years)	1.065	1.057-1.074	**<.001**	1.018	1.009-1.026	**<.001**
Female vs. male	0.972	0.836-1.130	.711	0.864	0.740-1.007	.062
pT3a vs. pT1b	1.753	1.461-2.105	**<.001**	1.491	1.239-1.794	**<.001**
N1 vs. N0	1.240	1.066-1.443	**.005**	1.206	1.019-1.428	**.029**
RCC type						
ccRCC	Ref.			Ref.		
pRCC	1.108	0.887-1.383	.386	1.010	0.805-1.267	.930
chRCC	0.565	0.378-0.843	**.005**	0.622	0.416-0.928	**.020**
Other	1.638	0.996-2.691	.052	1.737	1.055-2.861	**.030**
Tumor size (mm)	1.019	1.011-1.028	**<.001**	1.018	1.009-1.026	**<.001**
PN vs. RN	0.761	0.601-0.963	**.023**	0.906	0.708-1.159	.431

Significant *P*-values are given in bold.

HR, hazard ratio; PN, partial nephrectomy; Ref., reference category; RN, radical nephrectomy. ^a^Adjusted for: age at surgery (years), female vs. male sex, pT1b vs. upgraded pT3a, RCC type, tumor size (mm), and surgery PN vs. RN.

## Data Availability

The National Swedish Kidney Cancer Register (NSKCR) is an ongoing register and it is notavailable for open access.
